# Aneuploidy analysis in day 7 human blastocysts produced by in vitro fertilization

**DOI:** 10.1186/s12958-016-0157-x

**Published:** 2016-04-14

**Authors:** Yu Su, Jian-Jun Li, Cassie Wang, Ghassan Haddad, Wei-Hua Wang

**Affiliations:** Center for Reproductive Medicine, Changsha Hospital for Maternal and Child Health Care, Changsha, Hunan China; Vivere-Houston Fertility Laboratory, 2500 Fondren Road, Suite 350, Houston, TX USA; Houston Fertility Institute, Houston, TX USA; Vivere Health, Franklin, TN USA

**Keywords:** Aneuploidy, Delayed blastocyst, Implantation, PGS

## Abstract

**Background:**

Human embryos produced by in vitro fertilization (IVF) are usually cultured to day 6 after insemination, and good quality of embryos should develop to blastocyst stage at days 5 and 6. However, some embryos develop slowly, thus they may form blastocysts on day 7. Most IVF laboratories culture embryos to day 6 and then discard retarded embryos that do not develop to blastocyst stage. It has been reported that transfer of day 7 blastocysts can yield pregnancy although the rates were very low. In the present study, we evaluated the prevalence of aneuploidy in day 7 human blastocysts and also assessed embryo implantation after transfer of normal euploid blastocysts developed on day 7.

**Methods:**

Day 7 blastocysts were biopsied and screened for aneuploidy. Embryo implantation was assessed by transferring of euploid blastocysts.

**Results:**

A total of 1966 blastocysts from 367 IVF cycles were biopsied and screened for aneuploidy. It was found that 81.5 % of the patients had days 5 and 6 blastocysts and 18.5 % (68) patients had blastocysts developed on day 7, including 15.3 % had days 5–7 blastocysts and 3.3 % had only day 7 blastocysts. A total of 151 day 7 blastocysts, which accounted for 7.7 % of total blastocysts, were analyzed. It was found that 36.7 % of the blastocysts were euploid and 63.3 % had abnormal chromosomes, including aneuploidy and euploid with partial chromosome deletion. The aneuploidy rate was also maternal age dependent and was as high as 91.7 % in patients who were ≥40 years old. During the study period, transfer of day 7 euploid blastocysts in 15 patients resulted in 2 healthy live births.

**Conclusion(s):**

Aneuploidy rates in day 7 human blastocysts produced by IVF are very high. However, good euploid blastocysts have potential to implant and transfer of day 7 euploid blastocysts can result in healthy live birth. It is suggested that day 7 blastocyst culture may be necessary in patients who need aneuploidy screening.

## Background

Embryonic aneuploidy not only affects embryo development and implantation, but also increases miscarriage and birth defects [1–3]. The proportion of aneuploid embryos increases in women with aging [4–10]. Even in young patients undergoing in vitro fertilization (IVF), it has also been found that high proportions of embryos are aneuploid [11–14].

Preimplantation genetic screening (PGS) by DNA microarray and/or next-generation sequencing (NGS) has become an advanced technology in human IVF [7–10, 12–17]. Previously it has been found that PGS can increase clinical pregnancy and embryo implantation rates in patients of advanced maternal ages, recurrent miscarriage and previous spontaneous miscarriage, as aneuploidy is the main reason for unsuccessful embryo implantation in these populations of patients [9, 18–20]. As widespread application of PGS in human IVF clinics, it would appear that PGS may also increase embryo implantation rate in all patients undergoing IVF including young patients and recipients who receive egg donation [9, 10, 12, 14, 19], especially when single embryo transfer is performed [12, 14].

Human embryos are usually cultured up to day 6 after insemination, and blastocysts can be formed either on day 5 or day 6. However, slowly developed human embryos are occasionally observed and blastocysts are formed on day 7 in some patients [21–24]. Most IVF laboratories do not culture embryos to day 7 as day 7 blastocyst rate is low and blastocyst quality may not be as good as day 5 or day 6 blastocysts [21–26]. However, a few reports indicated that transfers of day 7 blastocysts can establish acceptable pregnancies [21, 25, 26] and suggested that embryos from some patients should be cultured up to day 7 [21].

PGS of human embryos have been performed on day 5 and day 6 blastocysts, and it has been reported there were not much differences between day 5 and day 6 blastocysts in terms of embryo quality and implantation [27, 28]. However, some reports found that day 6 blastocysts had lower implantation rates as compared with day 5 blastocysts [25, 29]. As far as we know, there is still no study or report to analyze day 7 human blastocysts in terms of aneuploidy formation and its relationship with embryo implantation. Therefore, in the present study, we retrospectively collected data on PGS of day 7 blastocysts derived from IVF patients in our clinic during 2014 and aimed to analyze whether a day 7 blastocyst culture is necessary for the patients who require PGS, and whether slowly developed human blastocysts have higher rate of aneuploidy.

## Methods

### Ethical statement

Patients undergoing IVF, embryo cryopreservation and PGS signed written consents for all laboratory and clinical procedures. The data was retrospectively collected from the medical records at the clinic during 2014 and the study was approved by New England Institutional Review Board (NEIRB 14–504).

### Patient stimulation and egg retrieval

Patients were stimulated with a combination of Follistim (Organo Inc, Roseland NJ, USA), Gonal-F (EMD Serono, Rockland MA, USA), Menopur (Ferring Pharmaceuticals, Parsippany NJ, USA) and/or Bravella (Ferring Pharmaceuticals) beginning 2–3 days after the onset of menses. The initial dose was 150–375 IU and was adjusted subsequently as the stimulation progressed. To prevent an LH surge, a GnRH antagonist, Ganirelix or Cetrorelix (Organo Inc.), was given when the leading follicle was 13–14 mm or when the estradiol level was 400 pg/ml. Human chorionic gonadotropin (hCG), Ovidrel (Serono USA), or a GnRH agonist, leuprolide acetate (Teva North America, North Wales PA, USA), was injected to induce final oocyte maturation when at least two dominant follicles reached a diameter of >18 mm. Eggs were retrieved under IV sedation via transvaginal ultrasound between 35–37 h after hCG or leuprolide acetate administration.

### Egg insemination, embryo culture and blastocyst biopsy

Matured eggs were inseminated by intracytoplasmic sperm injection (ICSI) 5–6 h after retrieval and inseminated eggs were cultured in Global medium supplemented with 10 % serum protein substitute (SPS) after ICSI. Fertilization was examined 16–18 h after ICSI and normally fertilized eggs (zygotes) were cultured in Global medium supplemented with 10 % SPS at 37 °C in a humidified atmosphere of 5.5 % CO_2_, 5 % O_2_ and balanced nitrogen until day 7 after inseminations.

At day 3, a hole about 20 μm was opened in the zona pellucida using the ZILOS-tk^TM^ laser system (Hamilton Thorn Bioscience Inc., MA USA). On day 5–7, embryos for biopsy were examined with an inverted microscope, and if the embryos developed to a full blastocyst stage and some trophectoderm (TE) cells started to hatch from the opening in the zona pellucida, some hatched TE cells (5 ~ 10) were biopsied using a 20 μm polished biopsy pipette with assisted cutting by the laser. Blastocyst biopsy was performed on TE cells at days 5, 6 and 7 depending on blastocyst development. After biopsy, the embryo proper was cultured in Global medium supplemented with 10 % SPS for 1–3 h before cryopreservation. The biopsied cells were washed with a washing buffer provided by PGS laboratories, placed in tubes with cell lysis buffer and were then frozen at −20 °C before being processed for microarray. Samples were analyzed with Illumina BluGnome 24Sure DNA microarray following the BluGnome 24Sure V3 protocol [9].

### Blastocyst vitrification, warming and embryo transfer

All blastocysts were vitrified after the blastocoele was completely collapsed by using Irvine vitrification kit (Irvine Scientific, Irvine, CA USA). Briefly, blastocysts were equilibrated in the warmed (37 °C) equilibration solution for 2 min and then transferred into the warmed (37 °C) vitrification solution (both steps were performed on a warming stage). The blastocysts were finally loaded onto a vitrification straw within 45 s. All embryos were vitrified individually and then stored in liquid nitrogen until warming for frozen embryo transfer (FET).

For warming, blastocysts were exposed to warming solution (Irvine warming kit) at 37 °C for 1 min. Blastocysts were then transferred to a dilution solution (0.5 M sucrose) for 3 min and then to a washing solution for 10 min with a solution change after 5 min at room temperature. After completion of the warming process, blastocysts were washed with Global medium supplemented with 10 % SPS and then cultured in the same medium for 2–16 h before transfer. Blastocyst quality was assessed using standard assessments developed by the Society for Assisted Reproductive Technologies [30].

### Patient preparation for frozen embryo transfer

All patients for embryo transfer received estradiol orally and transvaginally. Intramuscular administration of progesterone oil was initiated after about 14 days of estradiol treatment. Endometrium thickness was measured on the day of progesterone administration. Embryo transfer occurred on the sixth or seventh day of progesterone administration and progesterone was continued until the first serum β-hCG test two weeks after transfer. Ongoing pregnancies were supported by continued estradiol and progesterone.

### Pregnancy and implantation assessment

Fourteen days after embryo transfer, pregnancy was checked by a serum β-hCG assay. When the β-hCG was > 5 mIU/mL the patients were regarded as having a biochemical pregnancy. Four weeks after embryo transfer, when a gestational sac and a heart beat appeared ultrasonographically, the patients were diagnosed as having a clinical pregnancy.

## Results

**Fig. 1:**
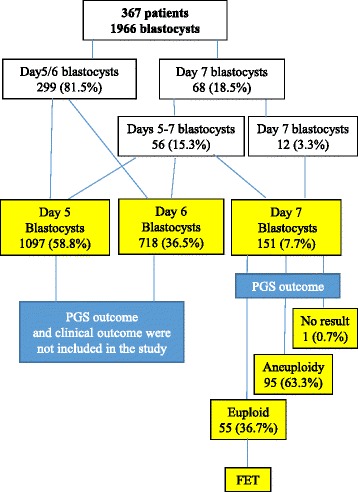
Diagraph of patient population, blastocyst development and embryo transfer (FET). Patients were grouped based on day 5/6 blastocyst, day 5–7 blastocyst and only day 7 blastocyst formation. Aneuploidy was analyzed only in day 7 blastocysts and clinical outcome was also analyzed based on day 7 euploid blastocyst transfer

As shown in Fig. 1, 1966 blastocysts from 367 patients were obtained in the present study. It was found that 81.5 % patients had day 5 and/or day 6 blastocysts and no blastocysts were observed on day 7. Sixty-eight patients, which accounted for 18.5 % of the total patients, had 151 blastocysts on day 7, which accounted for 7.7 % of the total blastocysts observed in the present study. Out of these 68 patients, 12 patients had only day 7 blastocysts, which accounted for 3.3 % of the total number of patients in the present study.

A total of 151 blastocysts at day 7 were biopsied and analyzed. As a result, 1 sample did not show test results due to poor quality or low quantity of DNA in the sample, 55 samples (36.7 %) had normal chromosomes (euploid) and 95 samples (63.3 %) had abnormal chromosomes including aneuploidy (91 samples, 95.8 %) and chromosomal microdeletion (4 samples, 4.2 %).

**Fig. 2:**
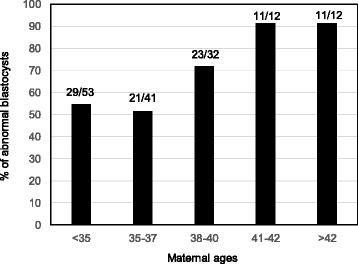
Abnormal day 7 blastocysts formation in patients at various maternal ages. Numbers in the top of each bar indicate the numbers of aneuploid blastocysts vs total number of blastocysts biopsied and screened

As shown in Fig. 2, aneuploid blastocyst rates were increased as maternal ages were increased from 54.7 % in the patients less than 35 years old to 91.6 % in the patients more than 40 years old.

In the present study, 56 patients had days 5–7 blastocysts but only 36 patients had euploid blastocysts and other 20 patients did not have any euploid embryos. For these patients who had day 5 and/or day 6 euploid blastocysts, day 5 and/or day 6 euploid blastocysts were preferentially transferred. As shown in Fig. 1, 12 patients had only day 7 blastocysts, but two patients did not have euploid blastocysts so embryo transfer was not performed.

As summarized in Table 1, 10 patients (number 1–10) had day 7 euploid blastocyst transfer and one patient was pregnancy after two good blastocysts were transferred. Finally, one embryo implanted and the patient delivered a healthy baby, while 9 patients were not pregnant after single embryo transfer and the quality of blastocysts is listed in Table 1. Three patients (number 11–13) were not pregnant after transfer of a day 5 or 6 euploid blastocyst, so each patient had a day 7 euploid blastocyst transfer, but embryos did not implant in all three patients (one patient had good embryos, but others had fair embryos). Two patients (number 14 and 15) had two mixed euploid blastocysts transferred. Two blastocysts (one from day 5 and another from day 7) were transferred in patient 14 and she was pregnant but was miscarried. Patient 15 also had two blastocysts transferred (one from day 6 and another from 7) and the patient was pregnant and delivered twins. Putting together, these data resulted in a 20 % (3/15) clinical pregnancy rate, a 18.8 % (3/16) implantation rate a 13.3 % (2/15) live birth rate and after transfer of day 7 euploid blastocysts in the present study.

**Table 1:** Clinical outcome in patients who received day 7 euploid blastocyst transfer

Case No.	Age	No. of day 5/6 euploid	No. of day 7 blastocysts	No. of day 7 euploid	No. of blastocysts transferred	Quality of blastocysts transferred^a^	Clinical pregnancy	No. of embryos implanted	No. of Live birth
1	34	0	3	1	1	Good/Good	No	0	0
2	33	0	2	2	1	Fair/Fair	No	0	0
3	37	0	1	1	1	Good/Good	No	0	0
4	39	0	2	1	1	Good/Fair	No	0	0
5	29	0	3	2	2	Good/Good	Yes	1	1
						Good/Good			
6	39	0	3	1	1	Fair/Fair	No	0	0
7	39	0	1	1	1	Fair/Fair	No	0	0
8	37	0	1	1	1	Fair/Fair	No	0	0
9	39	0	1	1	1	Fair/Fair	No	0	0
10	37	0	3	1	1	Poor/Poor	No	0	0
11^b^	36	1	2	1	1	Good/Good	No	0	0
12^b^	34	1	2	1	1	Fair/Fair	No	0	0
13^b^	29	1	1	1	1	Fair/Fair	No	0	0
14^e^	34	1	3	1	2^c^	Goog/Good	Yes	1^f^	0
						Good/Good			
15^e^	35	1	1	1	2^d^	Fair/Good	Yes	2^d^	2^d^
						Good/Fair			

^a^Quality of blastocyst: inner cell mass/trophectoderm

^b^Patients also had previous day 5 and/or day 6 euploid blastocyst transfer but embryos failed to implant

^c^One was day 5 blastocyst and another was day 7 blastocyst

^d^One was day 6 blastocyst and another was day 7 blastocyst

^e^Blastocysts from different culture days were transferred

^f^Patient was pregnant but was miscarried

## Discussion

The present study, as far as we know, is the first study to analyze aneuploidy formation in day 7 human blastocysts produced by IVF. Previously, although there are many publications to report aneuploidy formation in human blastocysts, all reports did not include day 7 blastocysts [7–13]. In the present study, we found that 63.3 % of day 7 blastocysts were aneuploid and the aneuploidy rate in day 7 blastocysts was also maternal age dependent, and was as high as 91.7 % in the patients at ages of 40 or more. We also found that 18.5 % of the patients had blastocysts at day 7 and 3.3 % of the patients had only day 7 blastocysts, i.e. no blastocysts at days 5 and 6. Although day 7 blastocysts accounted for only 7.7 % of the total blastocysts, and only 36.7 % of the day 7 blastocysts were euploid, transfer of these euploid blastocysts can successfully result in healthy live births, indicating that day 7 embryo culture may be necessary for the patients undergoing IVF, especially for those patients who do not have days 5 and/or 6 blastocysts.

More evidences indicate that there are more high quality of blastocysts on day 5 than day 6 or day 7, but the implantation rates were not different if same quality of blastocysts were transferred, irrespective of day 5 or 6 [27, 28]. However, if all blastocyst (various qualities) transfers at day 5 and day 6 were considered and compared, day 5 blastocysts had higher implantation rates [25, 29], indicating that quality of fast growing blastocysts is better than slow growing blastocysts. Same as the results in a previous study that days 5–7 blastocysts without doing PGS were transferred [21], we also found that both days 5 and 6 blastocysts had better quality than day 7 blastocysts (unpublished data). Because all of these embryos are euploid, thus the quality of embryos would be the key factor affecting the implantation. Lower implantation from day 7 euploid blastocysts may be mainly due to embryo quality [21]. Indeed, in the present study, we found that the quality of blastocysts transferred was the key factor affecting embryo implantation. No implantation was obtained if blastocysts had both fair ICM and TE. As shown in the Table 1, most day 7 blastocysts transferred had fair quality, thus it may be the reason that day 7 blastocysts had low implantation rate in the present study. We also observed low implantation rates when day 5/6 blastocysts with both fair ICM and TE were transferred (unpublished data).

Although only a small portion of IVF patients had day 7 blastocysts, or had only day 7 blastocysts, transfer of these day 7 euploid blastocysts can result in healthy births [21–24]. Thus it may be necessary to culture embryos to day 7 in all patients or in the patients who do not have blastocysts on days 5 and/or 6, irrespective of PGS or not. In the present study, we collected limited data during the study period and these results, together with previous studies without PGS [21–24], suggest that day 7 human embryo culture is necessary. However, we still need more data to demonstrate if fair or poor blastocysts obtained on day 7 need to be transferred or to be biopsied for PGS. These embryos may not be “strong” enough to undergo biopsy although biopsy of day 5/6 good blastocysts did not affect embryo implantation [31].

Clinical pregnancy rate observed in the present study was similar to those reported previously with day 7 blastocyst transfer without PGS [21, 24, 25]. It is difficult to compare the outcomes between studies to get the conclusion that PGS of day 7 blastocysts can improve embryo implantation. From the present study, we at least can conclude that transfer of day 7 blastocysts that have been biopsied for PGS can result in healthy live births.

There is still concern about the implantation rate of euploid blastocyst transfer in IVF patients. As of the data from published results, it would appear that the overall implantation rates were close to 50 % if euploid blastocysts were transferred. The reasons that other blastocysts still cannot implant after transfer are not known. The following factors have be considered for the low (not 100 %) implantation rates, such as embryo quality, transfer skills, uterine lining and acceptability of embryos by uterine. However, the trauma caused by biopsy and mosaicism of blastocysts must also be considered.

Blastocyst biopsy procedure is not non-invasive and is performed by laser assisted cutting. Isolation a few TE cells from a blastocyst may not affect the quality of the embryo [31]. However, this may be dependent up on the embryo quality and cell numbers in the embryos. If a good blastocyst is biopsied, such side effects may be small. However, if a fair or poor blastocyst is biopsied, it may cause trauma to the embryos, which in turn affects subsequent embryo implantation. Further studies are necessary to investigate the implantation of fair or poor blastocyst after biopsy. In addition, current PGS technology does not show mosaic status in the samples, thus it is difficult to state if a blastocyst is mosaic or not. Blastocyst mosaic is very common in human blastocysts [9, 11, 32], so the test result of euploid does not mean all cells in the remaining embryos have normal chromosomes. As reported by Haddad et al., transfer of a euploid blastocyst may have mosaic pregnancy [33].

Clinical application of PGS in human IVF has been increased recently since DNA microarray and NGS were introduced to analyze all chromosomes. Almost all practices have switched from cleavage embryo transfer to blastocyst transfer due to PGS procedures that need blastocyst biopsy and time-consuming DNA analysis. PGS procedures also requires that blastocysts are cryopreserved for future FET, especially in clinics where DNA analysis is not done in-house. Increase pregnancy and implantation rates have been reported after transfer of screened euploid blastocysts, especially in the patients with advanced maternal ages [9, 10, 19, 20]. However, as indicated in the previous reports [29, 30, 34–36], chromosomal mosaicism may cause incorrect embryo screening. Diploid-aneuploid mosaicism is the most common chromosome mosaic in human embryos [29, 37], thus some screened as abnormal embryos may have normal ICM, while some screened as normal embryos may have abnormal ICM [9, 32]. A few reports indicated that transfer of aneuploid blastocysts can result in birth of healthy babies [34–36].

According to the previous studies, high proportions of human blastocysts had chromosomes inconsistent between ICM and TE or among TE cells [9, 32]. These results indicated that blastocysts with abnormal chromosomes in their whole or partial TE cells may have euploid cells in their ICM [9, 32]. Patients may lose opportunity to get pregnant if these embryos are not transferred. As suggested by Liu et al. [9], it may be necessary to do a second biopsy for PGS if all embryos were diagnosed as abnormal in an IVF cycle, especially if the patients are young. However, according to Greco’s report [36], transfer of aneuploid embryos after additional consents were signed by the patients may be practical without a second biopsy that may cause more damages to embryos. As PGS is a cost IVF laboratory procedure, and biopsy of blastocysts is not non-invasive, its value to all patients, especially to young patients remains further investigation [38]. As indicated in the previous study by analysis of day 3 biopsy and PGS [39], multiple centers involved data analysis with large sample size may be necessary to demonstrate the significant effectiveness of current PGS in human IVF.

## Conclusions

In conclusion, our study indicates that aneuploidy rates are high in day 7 human blastocysts produced by IVF. However, euploid blastocysts can be found in day 7 blastocysts and transfer of these blastocysts can result in healthy live births. Some patients may have only day 7 blastocysts, thus 7 day embryo culture is necessary for these patients. Our results suggest that IVF practices may extend embryo culture to day 7 in the patients who do not have day 5 and/or day 6 blastocysts. While for the patients who have day 5 and/or day 6 blastocysts, day 7 culture may not be necessary after considering of the time and cost for the embryo culture, biopsy and genetic testing, as well as the low rates of good and euploid blastocysts. Further studies remain necessary to investigate the effectiveness of biopsy and PGS in fair and poor blastocysts as biopsy may negatively affect embryo’s capability to implant and blastocyst mosaic may reduce the available embryos for transfer.

## Abbreviations

IVF: in vitro fertilization
ICSI: intracytoplasmic sperm injection
PGS: preimplantation genetic screening
TE: trophectoderm
hCG: Human chorionic gonadotropin
ICM: Inner cell mass
NGS: next-generation sequencing
SPS: Serum protein substitute
FET: Frozen embryo transfer
